# Seasonality of Respiratory Viruses at Northern Latitudes

**DOI:** 10.1001/jamanetworkopen.2021.24650

**Published:** 2021-09-16

**Authors:** Michael T. Hawkes, Bonita E. Lee, Jamil N. Kanji, Nathan Zelyas, Kerry Wong, Michelle Barton, Shamir Mukhi, Joan L. Robinson

**Affiliations:** 1 Department of Pediatrics, University of Alberta, Edmonton, Alberta, Canada; 2 Department of Medical Microbiology and Immunology, University of Alberta, Edmonton, Alberta, Canada; 3 University of Alberta School of Public Health, Edmonton, Alberta, Canada; 4 Stollery Science Lab, University of Alberta, Edmonton, Alberta, Canada; 5 Women and Children’s Research Institute, University of Alberta, Edmonton, Alberta, Canada; 6 Public Health Laboratory, Alberta Precision Laboratories, University of Alberta Hospital, Edmonton, Alberta, Canada; 7 Division of Infectious Diseases, Department of Medicine, University of Alberta, Edmonton, Alberta, Canada; 8 Department of Laboratory Medicine and Pathology, Faculty of Medicine and Dentistry, University of Alberta, Edmonton, Alberta, Canada; 9 London Health Sciences Centre, Western University, London, Ontario, Canada; 10 Canadian Network for Public Health Intelligence, Edmonton, Alberta, Canada

## Abstract

**Importance:**

Every year, respiratory viruses exact a heavy burden on Canadian hospitals during winter months. Generalizable seasonal patterns of respiratory virus transmission may estimate the evolution of SARS-CoV-2 or other emerging pathogens.

**Objective:**

To describe the annual and biennial variation in respiratory virus seasonality in a northern climate.

**Design, Setting, and Participants:**

This cohort study is an epidemiological assessment using population-based surveillance of patients with medically attended respiratory tract infection from 2005 through 2017 in Alberta, Canada. Incident cases of respiratory virus infection and infant respiratory syncytial virus (RSV) hospitalizations in Alberta were extracted from the Data Integration for Alberta Laboratories platform and Alberta Health Services Discharge Abstract Database, respectively. A deterministic susceptible-infected-recovered-susceptible mathematical model with seasonal forcing function was fitted to the data for each virus. The possible future seasonal course of SARS-CoV-2 in northern latitudes was modeled on the basis of these observations. The analysis was conducted between December 15, 2020, and February 10, 2021.

**Exposures:**

Seasonal respiratory pathogens.

**Main Outcomes and Measures:**

Incidence (temporal pattern) of respiratory virus infections and RSV hospitalizations.

**Results:**

A total of 37 719 incident infections with RSV, human metapneumovirus, or human coronaviruses 229E, NL63, OC43, or HKU1 among 35 375 patients (18 069 [51.1%] male; median [interquartile range], 1.29 [0.42-12.2] years) were documented. A susceptible-infected-recovered-susceptible model mirrored the epidemiological data, including a striking biennial variation with alternating severe and mild winter peaks. Qualitative description of the model and numerical simulations showed that strong seasonal contact rate and temporary immunity lasting 6 to 12 months were sufficient to explain biennial seasonality in these various respiratory viruses. The seasonality of 10 212 hospitalizations among children younger than 5 years with RSV was also explored. The median (interquartile range) rate of hospitalizations per 1000 live births was 18.6 (17.6-19.9) and 11.0 (10.4-11.7) in alternating even (severe) and odd (less-severe) seasons, respectively (*P* = .001). The hazard of admission was higher for children born in severe (even) seasons compared with those born in less-severe (odd) seasons (hazard ratio, 1.68; 95% CI, 1.61-1.75; *P* < .001).

**Conclusions and Relevance:**

In this modeling study of respiratory viruses in Alberta, Canada, the seasonality followed a pattern estimated by simple mathematical models, which may be informative for anticipating future waves of pandemic SARS-CoV-2.

## Key Points

**Question:**

What is the seasonal pattern of respiratory viruses at northern latitudes?

**Findings:**

In this cohort study, a simple mathematical model was fitted to temporal data for 37 719 infections with respiratory syncytial virus, human metapneumovirus, or human coronaviruses 229E, NL63, OC43, or HKU1 and showed a marked biennial pattern. The same pattern was observed for 10 212 respiratory syncytial virus hospitalizations in young children.

**Meaning:**

These findings suggest that alternating severe and mild winter peaks occur with striking regularity for multiple virus species, providing a pattern of health care utilization and possibly anticipating the evolution of the SARS-CoV-2 pandemic.

## Teaser

This cohort study describes the annual and biennial variation in respiratory virus seasonality in a northern climate.

## Introduction

Seasonal epidemics of viral respiratory tract infections strike with clocklike regularity during winter in temperate zones. Infections with these viruses cause self-limited disease in healthy hosts but can cause significant morbidity and mortality in susceptible individuals.^1^ Common circulating respiratory viruses include respiratory syncytial virus (RSV), human metapneumovirus (hMPV), and seasonal human coronaviruses (HCoVs), including the alphacoronaviruses (HCoV-229E and HCoV-NL63) and betacoronaviruses (HCoV-OC43 and HCoV-HKU1). Understanding the strong seasonal pattern of respiratory viruses may help anticipate subsequent waves of SARS-CoV-2.

Alternating cycles of large and smaller annual epidemics of respiratory viruses are observed in some countries.^2,3,4^ This biennial pattern is accentuated at northern latitudes and is best described for RSV.^2^ Previous authors have shown that simple mathematical models can estimate this phenomenon,^2^ which results from a period-doubling bifurcation when seasonality is strong, creating alternating years of high and low prevalence.^5^

The objective of this study was to examine the seasonal variation of infections with RSV, hMPV, and HCoVs and of RSV hospitalizations in Alberta, Canada. A simple mathematical model was fitted to observed data. Implications of this work include generalizable estimations of respiratory virus epidemiology in northern zones, which may have value for anticipating RSV hospitalizations and the evolution of the SARS-CoV-2 pandemic and future emerging pathogens.

## Methods

Approval for this cohort study was obtained from the University of Alberta Health Research Ethics Board. Deidentified data were obtained from a laboratory database; therefore, informed consent was not obtained from each individual patient, per University of Alberta policy. Our study followed standard reporting guidelines for modeling studies.^6^

### Study Setting and Data Sources

Alberta (population 4.3 million individuals,^7^ latitude 49°N to 60°N) has a central provincial laboratory (Alberta Precision Laboratories–Provincial Laboratory) that processed all respiratory virus samples. Data for all testing were derived from Data Integration for Alberta Laboratories.^8^ Data were cleaned to remove samples from the same patient yielding the same virus within 30 days because this was assumed to be a single infection. Indications for obtaining a respiratory tract specimen were based on the judgment of the submitting clinician. Testing algorithms and platforms varied over time (eAppendix 1 in the Supplement).

Data for inpatients with RSV were derived from the Alberta Health Services Discharge Abstract Database, which captures discharge diagnoses from all hospitals in the province. Using a validated search strategy,^9^ we identified all children (aged 0-5 years) admitted to any hospital in Alberta with a primary diagnosis of RSV from July 1, 2004, until June 30, 2017 (*International Statistical Classification of Diseases and Related Health Problems, Tenth Revision* codes J12.1, J20.5, J21.0, or B97.4).

### Mathematical Epidemiological Model

**Figure 1.**
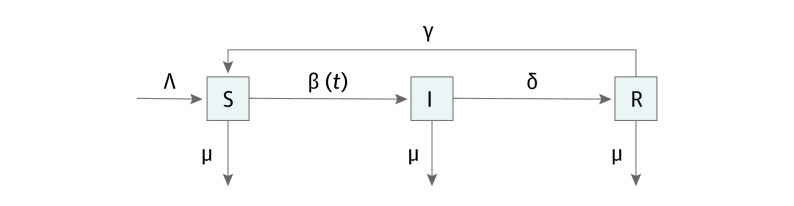
Susceptible (S) Infected (I) Recovered (R) Susceptible Epidemiological Model Flow Diagram The variables used in the model include births (Λ), natural deaths (μ), contact rate as an annual periodic function (β(*t*)), duration of infection (approximately 1/δ), and duration of immunity (approximately 1/γ).

We used a deterministic susceptible-infected-recovered-susceptible (SIRS) compartmental model, with waning immunity (Figure 1).^10^ This standard model^11^ has previously been used to model RSV.^2^ The model takes into account vital dynamics (births [Λ] and natural deaths [μ]), contact rate as an annual periodic function (β(*t*)), duration of infection (approximately 1/δ), and duration of immunity (approximately 1/γ).

A system of nonlinear ordinary differential equations describes the flow between compartments:
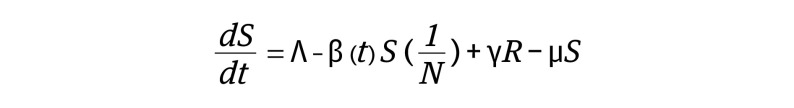
,

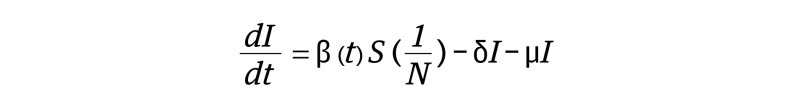
, and

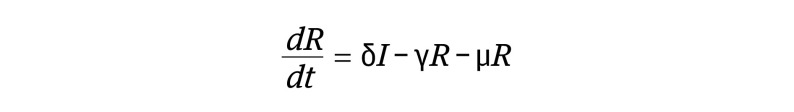
,where β(*t*) includes seasonal forcing:
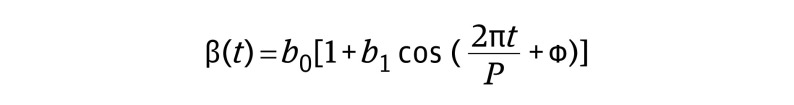
.The contact rate is modeled as a cosine function, with mean contact rate *b*_0_, amplitude *b*_1_, period *P* = 1 year, and phase shift ϕ.

### Definitions

Respiratory virus season was defined as beginning on July 1 and ending on June 30 of the subsequent year. Even seasons were defined as the period beginning in an even-numbered year (eg, July 1, 2012, to June 30, 2013). Odd seasons were defined as the period beginning in an odd-numbered year (eg, July 1, 2013, to June 30, 2014).

### Parameter Estimates

Biological properties of respiratory viruses that inform the parameter estimates are reviewed in eAppendix 2 in the Supplement. For numerical simulations of solutions to the ordinary differential equations, we used the Euler method in the R statistical environment (R statistical software version 3.6.2; R Project for Statistical Computing).^12^ We used the steady-state endemic equilibrium as initial conditions and a run-in period of 50 years to model the stable limit cycle. The optimal parameter fits were found by minimizing the sum of squared residuals between the model estimates of weekly disease incidence and the observed weekly cases detected (ordinary least squares regression). We assumed that weekly cases detected represented a proxy of the incidence of disease in the population, related through a constant scaling factor (*s*). During model fitting, the values of 3 parameters based on vital statistics (*N*, Λ, and μ)^13^ were held constant at the values given in Table 1. Values of 6 parameters (*s*, ϕ, *b*_0_, *b*_1_, δ, and γ) were varied iteratively, rerunning the numerical simulation with each parameter set until a minimum sum of squared residuals was reached. A local minimum was demonstrated by graphical methods.^14^ The quality of the fit was expressed as the standard error (root mean square of residuals).

**Table 1.** Model Parameters: Values and Rationale

Parameter	Estimate	Comments	Reference
Fixed^a^			
* N*	4 428 247	Population: population of Alberta (2020)	Vital statistics^13^
Λ	137 d^−1^	Birth rate: 52 334 births in Alberta (2018); approximated as μ × *N* as a simplifying assumption of constant population size	Vital statistics^13^
μ	0.0000319 d^−1^	Natural mortality rate (population): 25 990 deaths in Alberta (2018)	Vital statistics^13^
Fitted^b^			
*b*_0_	0.121 d^−1^	Average of transmission parameter, β(t): related to basic reproduction number	Weber et al^2^
*b*_1_	0.249	Amplitude of seasonal variation in transmission parameter, β(t): higher values at northern latitudes (eg, 0.36 Finland vs 0.10 Florida)	Weber et al^2^
δ	0.0986 d^−1^	Rate of loss of infectiousness: related to the mean duration of infection: approximately 10 d	Weber et al^2^
γ	0.00469 d^−1^	Rate of loss of immunity: related to the mean duration of immunity: approximately 200 d	Weber et al^2^
* s*	1/2000	Scaling factor: optimal value chosen to match model endemic equilibrium to mean weekly number of cases	Weber et al^2^
ϕ	0	Phase shift: optimal value chosen to match timing of seasonal peaks	Weber et al^2^

^a^ Parameters are based on vital statistics and were held constant during model fitting.

^b^ Initial estimates (shown in table) were optimized for each virus, using ordinary least squares regression to fit model to epidemiological data.

### Statistical Analysis

Descriptive statistics included median and interquartile range (IQR) for continuous variables and number (percentage) for categorical variables. To examine associations between continuous variables, nonparametric methods (Mann-Whitney *U* test) were used. *P* values were 2-sided, and statistical significance was set at *P* = .05. A multivariable Cox proportional hazard model was used to examine the impact of season of birth (odd vs even) on the hazard of hospitalization for RSV. Members of the birth cohort who were not hospitalized for RSV were censored at age 5 years. Month of birth was entered in the model as a categorical covariate (12 level factor). Further details of this model are provided in the eAppendix 3 in the Supplement. The package survival^15^ in the R statistical environment (R software version 3.6.2)^12^ was used. Data analysis was conducted from December 15, 2020, to February 10, 2021.

## Results

### Biennial Pattern in the Incidence of Respiratory Virus Infection

From November 2005 to April 2017, 282 386 samples were received at Alberta Precision Laboratories–Provincial Laboratory for respiratory virus testing. Of those specimens, 40 560 (14.4%) received from 35 375 patients (18 069 [51.1%] male; median [IQR] age, 1.29 [0.42-12.2] years) tested positive for 1 or more of RSV, hMPV, HCoV 229E, HCoV NL63, HCoV HKU1, and HCoV OC43 and were included in the study. A total of 2857 specimens with the same virus detected within 30 days from the same patient were removed as duplicate specimens. The remaining 37 719 positive specimens were considered to represent incident cases of respiratory virus infection. Characteristics of patients and seasonal epidemics are shown in Table 2.

**Table 2.** Characteristics of Patient and Seasonal Epidemics of Respiratory Viruses in Alberta, Canada, 2005-2013

Characteristic	Median (IQR)					
	RSV	hMPV	HCoV			
			229E	NL63	OC43	HKU1
Patients						
No. of patients	24 336	8647	1091	1523	1385	627
Total No. of specimens	24 962	8774	1105	1540	1395	633
Age, y	0.990 (0.330-2.78)	3.23 (0.930-49.0)	32.3 (2.65-55.3)	2.24 (0.575-35.1)	13.2 (0.84-65.5)	4.78 (0.792-46.9)
Sex, No. (%)^a^						
Female	11 595 (47.1)	4314 (50.0)	544 (49.5)	718 (47.0)	702 (50.5)	302 (48.6)
Male	12 741 (52.9)	4333 (50.0)	547 (50.5)	805 (53.0)	683 (49.5)	325 (51.4)
Seasonal epidemics						
No. of cases per season						
Odd	1340 (1310-1610)	1130 (962-1360)	159 (141-159)	78.0 (58.2-109)	52.5 (34.8-70.7)	120 (112-202)
Even	2600 (2500-2900)	423 (331-460)	32.8 (26.5-86.8)	245 (207-258)	204 (198-262)	15.6 (12.0-16.5)
Ratio of high to low incidence seasons	1.86 (1.62-1.9)	2.66 (2.61-2.99)	5.68 (4.46-6.45)	3.27 (2.6-4.65)	3.68 (3.32-6.7)	7.5 (6.81-21.5)
Mean weekly cases (overall)	41.3 (27.1-49.5)	12.4 (8.31-20.9)	2.37 (0.635-3.06)	2.69 (1.92-4.71)	1.67 (1.01-3.81)	1.16 (0.312-2.23)
Model fit, SE of model, cases/wk	23.0	12.0	3.93	3.49	4.33	3.06

Abbreviations: HCoV, human coronavirus; hMPV, human metapneumovirus; IQR, interquartile range; RSV respiratory syncytial virus.

^a^ Sex was unknown for 495 patients (1.3%).

**Figure 2.**
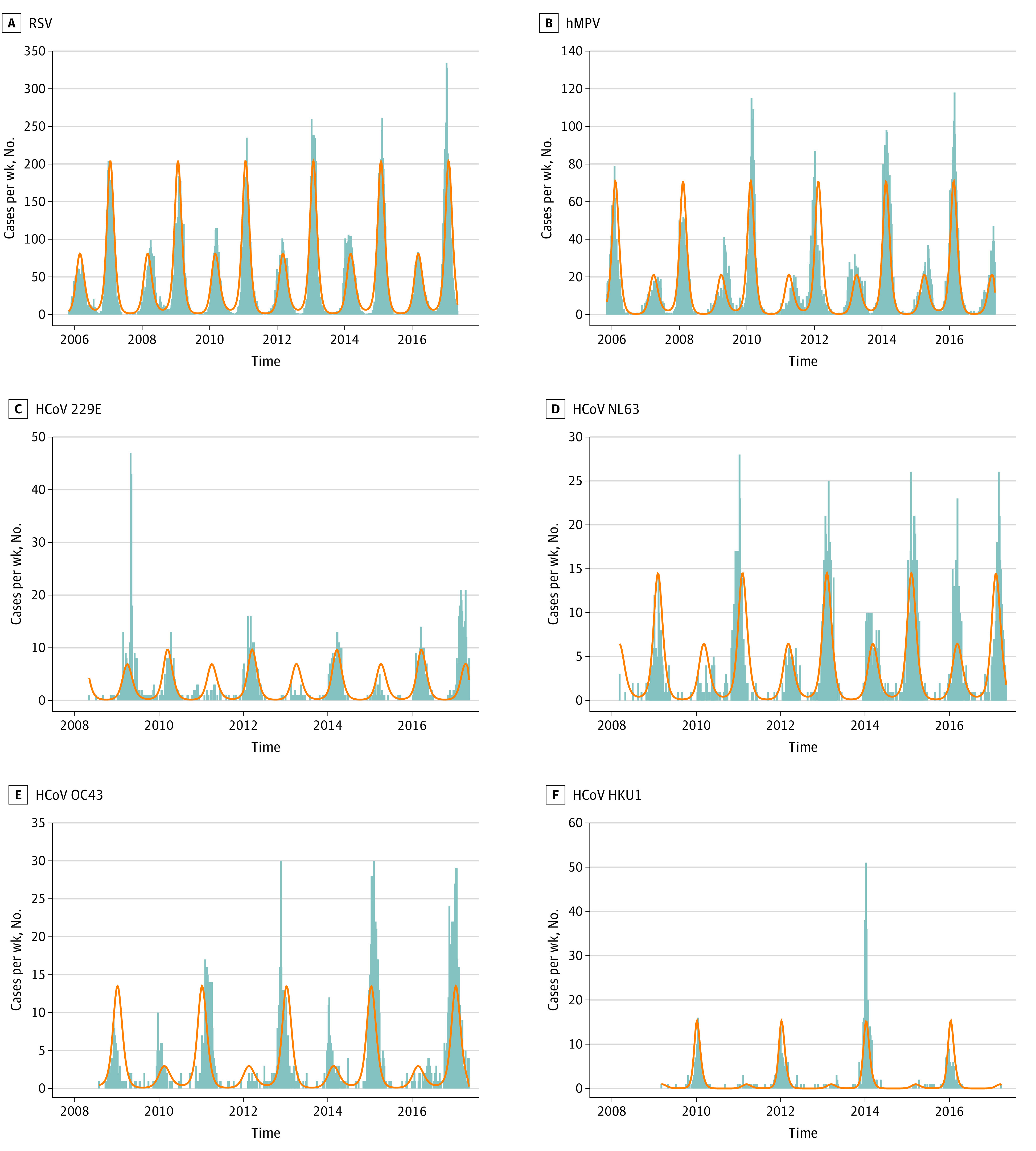
Seasonal Epidemics of Selected Respiratory Viruses in Alberta, Canada, 2005-2017 The weekly number of cases (gray bars) detected in the province showed a biennial pattern with alternating high incidence and low incidence seasons. The incidence was accurately modeled with a susceptible-infected-recovered-susceptible model incorporating strong seasonality and temporary protective immunity (orange line). Carry-over of herd immunity from a high-incidence season may explain low incidence in the subsequent season. A, Respiratory syncytial virus (RSV) was the most frequently detected virus (mean [SD], 41.3 [13.0] cases/week) with highest incidence in even seasons (eg, 2010-2011). B, Human metapneumovirus (hMPV) incidence (mean [SE], 12.4 [8.2] cases/week) was highest in odd seasons. C, Human coronavirus (HCoV) 229E was less frequently detected (mean [SE], 2.37 [1.57] cases/week) and exhibited stochastic variation. Incidence was highest in odd seasons, although 1 week of exceptionally high incidence occurred in 2009 and a high incidence season occurred in 2016 to 2017. D, HCoV NL63 incidence (mean [SE], 2.69 [1.75] cases/week) was highest in even seasons. Exceptionally high incidence was observed in 2015 to 2016. E, HCoV OC43 incidence (mean [SE], 1.67 [2.10] cases/week) was highest in even seasons. One week of high activity was noted in 2012. B, HCoV HKU1 incidence (mean [SE], 1.16 [2.04] cases/week) was highest in odd seasons and cases were nearly absent in alternating even seasons. Several weeks of exceptional activity were noted in 2014.

A SIRS model with seasonal forcing explained the epidemiological data with surprising accuracy (Table 2). A qualitative description of the model and the mathematical basis of the biennial seasonality is provided in eAppendix 4, eFigure 1, eFigure 2, and eFigure 3 in the Supplement. The fitted parameters are shown in the eTable in the Supplement, with graphical demonstration of parameter optimization shown in eFigure 4, eFigure 5, eFigure 6, eFigure 7, eFigure 8, and eFigure 9 in the Supplement. We explored the conditions necessary to generate a biennial pattern in the SIRS model by varying key model parameters *b*_1_ and γ. A biennial pattern emerged when the seasonality was intense (*b*_1_ > 0.17; eFigure 3A in the Supplement). Furthermore, the biennial pattern was observed over a limited range of values of duration of immunity: 
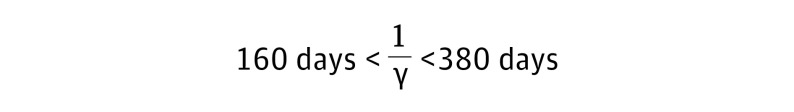
as shown in eFigure 3B in the Supplement. Characteristics of annual virus epidemics are shown in Figure 2 and clearly show an alternating pattern of severe and less severe seasons, with higher number of cases, higher peak weekly cases, and earlier onset during severe seasons.

### Implications of Biennial Seasonality

#### RSV Hospitalizations

**Figure 3.**
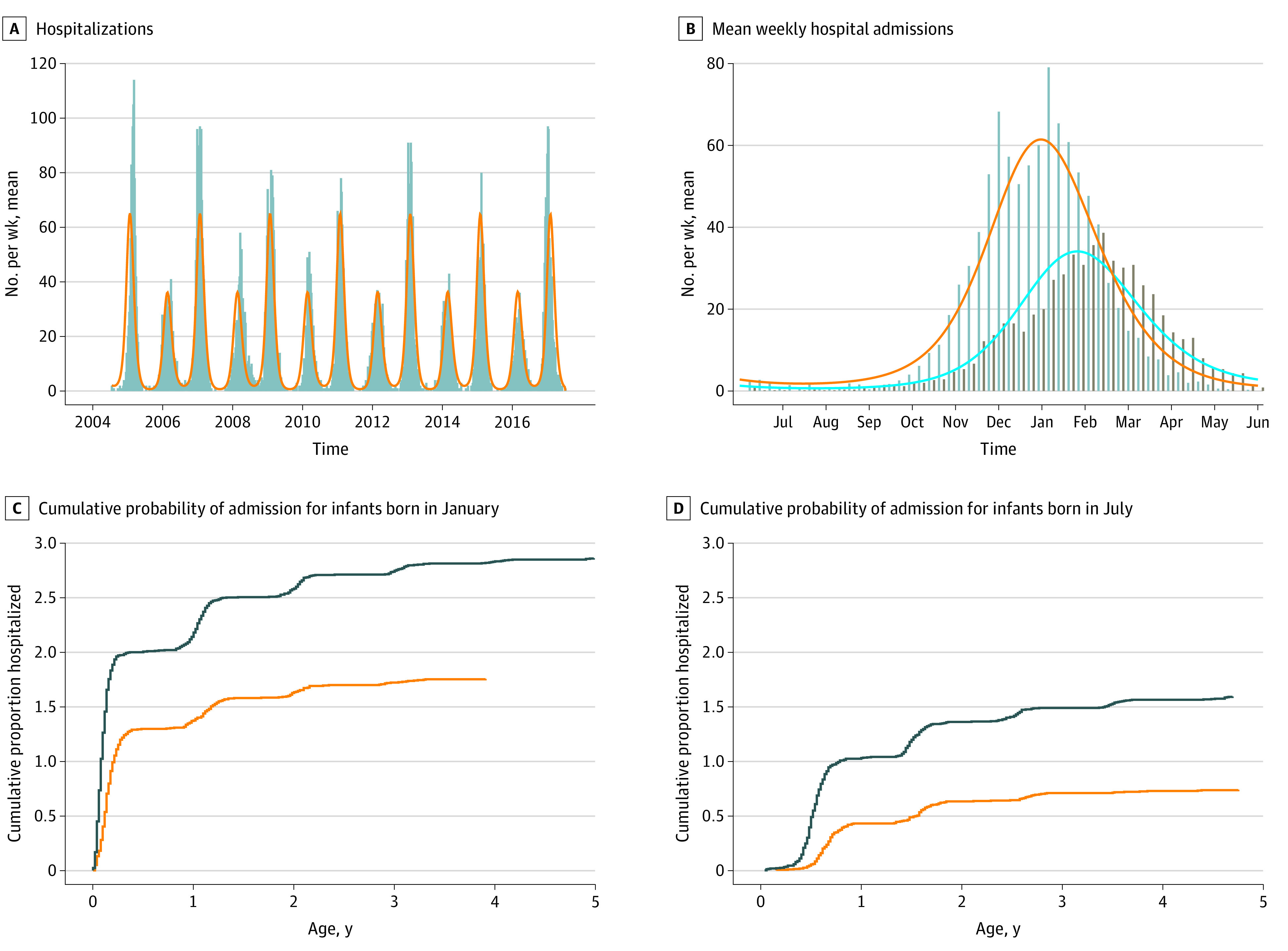
Hospitalizations for Respiratory Syncytial Virus (RSV) in Alberta, Canada, 2004-2017 A, Weekly hospitalizations (gray bars) and fitted mathematical model (orange line) show biennial pattern. B, Mean weekly admissions in even respiratory virus seasons (gray bars, orange line) show higher and earlier peak, compared with odd seasons (brown bars, blue line), coinciding with laboratory-based data (Figure 2A). C, Cumulative probability of admission for infants born in January of even (black line) and odd (orange line) seasons. Early infancy during the RSV season was associated with high admission rates in the first months of life, with plateau during the first summer of life and another wave of cases in subsequent winters. Infants born in January of even seasons had a higher hazard of admission over the first 5 years of life. D, Cumulative probability of admission for infants born in July of even (black line) and odd (orange line) seasons. Born in a summer month, the rate of hospitalization was lower in early infancy, with waves of admissions during the first and subsequent winters. Overall, fewer July-born infants were hospitalized with RSV before their fifth birthday than January-born infants, and the risk of hospitalization was lower among infants born in odd seasons.

From July 1, 2004 to June 30, 2017, 10 212 children younger than 5 years were admitted with RSV. As with the weekly virus detected, a biennial seasonal pattern was observed (Figure 3). The median (IQR) rate of hospitalizations per 1000 live births was 18.6 (17.6-19.9) and 11.0 (10.4-11.7) in alternating years (*P* = .001).

The model estimates that children born in severe RSV seasons would have a higher risk of hospitalization than children born in less-severe RSV seasons, an estimation that was substantiated by our data (Figure 3C and 3D). Fitting a Cox proportional hazard model to the observed data, we estimated that the hazard of admission was higher for children born in severe (ie, even) respiratory virus seasons compared with those born in less-severe (ie, odd) seasons (hazard ratio, 1.68; 95% CI, 1.61-1.75; *P* < .001).

#### Future Waves of SARS-CoV-2

Given the accuracy of the SIRS model for modeling the seasonal epidemics of several respiratory viruses, we speculated that the model could be used to estimate future waves of SARS-CoV-2. Several possible trajectories were modeled, including no seasonality (stable endemic equilibrium), regular seasonal peaks, and biennial pattern (eFigure 1 in the Supplement). If SARS-CoV-2 demonstrates seasonality similar to that of other respiratory viruses at northern latitudes, repeated winter outbreaks may be anticipated, with high incidence seasons alternating with lower incidence seasons.

## Discussion

The findings of this cohort study suggest that the biennial variation of several respiratory viruses is a general phenomenon, estimated by a simple mathematical model that depends only on strong seasonality and transient protective immunity. We were able to model the weekly counts of laboratory-confirmed RSV, hMPV, and HCoVs in our cohort with a parsimonious model. This modeling work confirms and extends previous findings on RSV seasonality,^2,5,16^ demonstrating model fit to other respiratory viruses, exploring the conditions under which biennial peaks occur, showing the implications for infant hospitalizations for RSV, and estimating future seasonal patterns of SARS-CoV-2.

For multiple respiratory viruses, at northern latitudes, seasonal oscillations in the infectious fraction show a distinct pattern, with a secondary maximum every second year.^2,17^ This intriguing dynamic behavior is well-explained by the SIRS model (eFigure 1 in the Supplement). In particular, the biennial variation is explained by a period-doubling bifurcation in the limit cycle. This phenomenon would only be observed under extreme seasonality, at northern latitudes, as in Turku, Finland,^2,5^ and in our setting. The biennial pattern also depends on the duration of immunity, arising when immunity wanes over 160 to 380 days (eFigure 3 in the Supplement). With respect to seasonal HCoVs, observational studies and experimental studies^13,18,19,20^ of healthy volunteers showed that the duration of natural immunity declines over approximately 1 year, consistent with our mathematical model. There is a paucity of information about the longevity of immunity to SARS-CoV-2^21^; however, primary infection appears to provide short-term immunity.^22^ Thus, the epidemiological characteristics required for biennial seasonality may be common to SARS-CoV-2 and other coronaviruses (eAppendix 4 in the Supplement). We speculate that, under natural conditions, seasonal outbreaks of SARS-CoV-2 in northern climates may follow the same biennial pattern. Large-scale vaccination campaigns and other public health measures could alter this anticipated pattern.

Explanatory mechanisms for seasonality of respiratory viruses include factors that affect host contact rates, virus survival in the environment, and host immunity.^23^ In our mathematical model, these mechanisms contribute collectively to a seasonal forcing function, which drives periodic transmission. For RSV, strong seasonality leads to annual strain on health care facilities in winter months due to high numbers of hospitalized infants. Moreover, the biennial seasonality gives rise to a remarkable finding that month and year of birth are associated with RSV hospitalization in Alberta. For example, a child who happens to be born in a high-burden (even) RSV season has a hazard of hospitalization 1.68-fold higher than that of a child born in an odd season (Figure 3).

The implication of these findings for the SARS-CoV-2 pandemic in the Northern Hemisphere are worth considering. The winter 2020 to 2021 SARS-CoV-2 epidemic across Canada threatened to overwhelm health system capacity and required renewed intensive public health measures. The natural fluctuation in case counts may have given the appearance that SARS-CoV-2 was successfully contained in spring and summer, with loss of control of the epidemic in the winter. Beyond its first winter, recurrent waves of SARS-CoV-2 of variable magnitude may occur, as with influenza A virus (H1N1).^24^ On the other hand, although other HCoVs predominate in winter, SARS-CoV-2 appears to transmit efficiently in tropical climates.^25,26^ Therefore, cold and dry conditions are not a necessary condition of SARS-CoV-2 spread. Another study^27^ has incorporated seasonality in a SIR model of SARS-CoV-2, predicting prolonged and recurrent pandemic waves. We used our model to speculate on future SARS-CoV-2 seasonal outbreaks, showing that plausible assumptions about the duration of immunity would anticipate a seasonal pattern similar to those observed for other respiratory viruses, with biennial variation in peak incidence.

### Limitations

Limitations of our study include the use of laboratory-based data and administrative data on RSV hospitalizations. Medically attended illness of sufficient severity to warrant a nasopharyngeal swab was necessary for infections to be captured in the laboratory database. Likewise, hospitalization for RSV is dependent on patient age and comorbidities. Ideally, we would have included data on RSV hospitalization among elderly patients but RSV infection in this population is underrecognized, so testing is often not performed. Although the number of positive laboratory tests is not a direct measure of incidence in the community, it is a reasonable proxy that we expect to be correlated. With respect to our mathematical modeling strategy, our objective was to explore a simple but versatile model that might be applied to several respiratory viruses. Although more complex models may provide more accurate estimates, they depend on correct parameters, which must be derived from large and multiple data sets for the specific context. Simple models avoid detailed assumptions and can be interrogated to investigate the effects of an aspect of disease such as seasonality. Numerous assumptions were made, including perfect mixing, no in-migration or out-migration of infected individuals, exponential distribution of the duration of infection and immunity, and immunity conceptualized as a binary state. We relied on both numerical simulations and qualitative analysis of the system of nonlinear, nonautonomous ordinary differential equations to describe the rich dynamic behavior because there was no analytical solution to the model.^5^

## Conclusions

In summary, in this cohort study, many respiratory viruses in northern zones exhibited a biennial seasonal pattern. The winter wave of SARS-CoV-2 that overwhelmed hospital surge capacity across Canada may follow a similar pattern in the absence of large-scale vaccination programs. On the basis of lessons learned from past respiratory virus seasons in Canada, simple deterministic models may have explanatory power in anticipating the future course of the pandemic and future emerging pathogens at northern latitudes.

## Notes

Supplement. **eAppendix 1.** Testing Algorithms and Platforms for Detection of Respiratory Viruses **eAppendix 2.** Properties of Respiratory Viruses That Inform Model Parametrization **eAppendix 3.** Cox Proportional Hazard Model for Time to First RSV Hospitalization **eAppendix 4.** Qualitative Analysis of Model **eFigure 1.** SIRS Mathematical Model of Seasonal Respiratory Viruses **eFigure 2.** Stable Biennial Limit Cycle **eFigure 3.** Sensitivity Analysis and Bifurcation Diagrams, Showing Model Predictions With Different Seasonality (*b_1_*) and Duration of Immunity (γ) **eTable.** Model Parameters That Optimize the Fit Between SIRS Model and Observed Weekly Counts of Respiratory Viruses **eFigure 4.** Optimization of Model Parameters for RSV **eFigure 5.** Optimization of Model Parameters for hMPV **eFigure 6.** Optimization of Model Parameters for HCoV 229E **eFigure 7.** Optimization of Model Parameters for HCoV NL63 **eFigure 8.** Optimization of Model Parameters for HCoV OC43 **eFigure 9.** Optimization of Model Parameters for HCoV HKU1
eReferences
