# Supplementary material for: Seasonality of Respiratory Viruses at Northern Latitudes
Source: JAMA Netw Open. 2021 Sep 16;4(9):e2124650. doi: 10.1001/jamanetworkopen.2021.24650 (PMC8446819; doi:10.1001/jamanetworkopen.2021.24650)
Supplement: Supplement. — eAppendix 1. Testing Algorithms and Platforms for Detection of Respiratory Viruses eAppendix 2. Properties of Respiratory Viruses That Inform Model Parametrization eAppendix 3. Cox Proportional Hazard Model for Time to First RSV Hospitalization eAppendix 4. Qualitative Analysis of Model eFigure 1. SIRS Mathematical Model of Seasonal Respiratory Viruses eFigure 2. Stable Biennial Limit Cycle eFigure 3. Sensitivity Analysis and Bifurcation Diagrams, Showing Model Predictions With Different Seasonality (b1) and Duration of Immunity (γ) eTable. Model Parameters That Optimize the Fit Between SIRS Model and Observed Weekly Counts of Respiratory Viruses eFigure 4. Optimization of Model Parameters for RSV eFigure 5. Optimization of Model Parameters for hMPV eFigure 6. Optimization of Model Parameters for HCoV 229E eFigure 7. Optimization of Model Parameters for HCoV NL63 eFigure 8. Optimization of Model Parameters for HCoV OC43 eFigure 9. Optimization of Model Parameters for HCoV HKU1 eReferences [file jamanetwopen-e2124650-s001.pdf]

Supplemental Online Content

Hawkes MT, Lee BE, Kanji JN, et al. Seasonality of respiratory viruses at northern latitudes. *JAMA Netw Open*. 2021;4(9):e2124650.
doi:10.1001/jamanetworkopen.2021.24650

eAppendix 1. Testing Algorithms and Platforms for Detection of Respiratory Viruses
eAppendix 2. Properties of Respiratory Viruses That Inform Model Parametrization
eAppendix 3. Cox Proportional Hazard Model for Time to First RSV Hospitalization
eAppendix 4. Qualitative Analysis of Model
eFigure 1. *SIRS* Mathematical Model of Seasonal Respiratory Viruses
eFigure 2. Stable Biennial Limit Cycle
eFigure 3. Sensitivity Analysis and Bifurcation Diagrams, Showing Model Predictions With Different Seasonality (b_1) and Duration of Immunity (γ)
eTable. Model Parameters That Optimize the Fit Between SIRS Model and Observed Weekly Counts of Respiratory Viruses
eFigure 4. Optimization of Model Parameters for RSV
eFigure 5. Optimization of Model Parameters for hMPV
eFigure 6. Optimization of Model Parameters for HCoV 229E
eFigure 7. Optimization of Model Parameters for HCoV NL63
eFigure 8. Optimization of Model Parameters for HCoV OC43
eFigure 9. Optimization of Model Parameters for HCoV HKU1
eReferences

This supplemental material has been provided by the authors to give readers additional information about their work.

© 2021 Hawkes MT et al. *JAMA Network Open.*

eAppendix 1. Testing Algorithms and Platforms for Detection of Respiratory Viruses

Testing algorithms and platforms varied over time as follows: From January 2009 to March 2009, all nasopharyngeal (NP) samples were first subjected to direct immunofluorescence antigen (DFA) test using monoclonal antibodies from Imagen (Lenexa, KS) for RSV, influenza A and B and parainfluenza virus and DFA-negative NP specimens and all other respiratory samples were tested using xTAG respiratory viral panel (RVP) from Luminex Molecular Diagnostics (Toronto, Ontario, Canada). Starting in March 2009, all respiratory specimens, regardless of DFA results, were tested using an in-house influenza A and B nucleic acid amplification test and RVP. Starting on June 19, 2009, RVP was performed only when respiratory specimens tested negative for influenza using the in-house assay for patients seen at emergency departments, hospitalized patients, provincial influenza-like-illness community surveillance program, and health regions where all specimens were transferred from health centers with no differentiation between specimens from the community versus acute care sites. (26).

© 2021 Hawkes MT et al. *JAMA Network Open.*

eAppendix 2. Properties of Respiratory Viruses That Inform Model Parametrization

Properties of Respiratory syncytial virus (RSV)

RSV infection is nearly ubiquitous in infancy, and most children are infected by the age of 2 years (1). In Alberta, approximately 1.6% of children have one or more RSV hospitalizations before the age of 5 years, with most infections occurring in the first year of life (2).

RSV shows striking seasonality, which is accentuated in northern latitudes (3). In temperate zones, most RSV cases occur in the cold season and in tropical zones, most cases occur in the wet season (4). The mean duration of viral shedding is 6.7 days (range 1-21) (5). Re-infection is common, with annual re-infection rates of 20%, 17%, 10%, and 3-6% among children 5-9, 10-14, 15-19 years of age, and adults, respectively (6). Immunity wanes over time, as demonstrated experimentally in adults with previous natural immunity to RSV re-challenged with a homologous RSV strain. By 2 months after natural RSV infection, 50% of healthy adults became re-infected, and by 8 months, two thirds became re-infected (7). Within 26 months, 76% had two or more infections and 47% had three or more infections (7). Most RSV infections are mild, even in young infants. The rate of hospitalization among infected infants is 1.6 to 3.3% (1, 8).

Properties of human metapneumovirus (hMPV)

Nearly all individuals are infected with hMPV early in life. One quarter of Dutch children between 6 and 12 months of age and nearly all five-year-old children had antibodies to hMPV (9). Similarly, in a Japanese cohort, 77% and 100% of children 2 to 5 and >10 years of age had hMPV antibodies, respectively (10). In a study from Thailand, 99.7% of children 6-16 years old had positive hMPV serology (11). Four genetic subgroups of hMPV (A1, A2,

© 2021 Hawkes MT et al. *JAMA Network Open.*

B1, and B2) are recognized, with variable incidence from year to year (12). They are antigenically similar and induce a variable cross-neutralizing antibody response (12, 13).

Reinfection occurs throughout childhood. In Thailand, 5% of children had evidence of reinfection over a four-year period, as defined by a 4-fold rise in immunoglobulin titres. Many children with acute hMPV exhibit both a positive IgM and IgG at presentation, followed by a 4-fold rise in IgG, indicating that they were previously infected, but that circulating IgG does not confer complete immunity (10). In non-human primate models, antibodies can mediate protection but titers wane over time (14). Taken together, these results suggest that transient protective immunity to hMPV allows recurrent infection over the lifetime of an individual.

Seasonality of hMPV is well documented in temperate zones. A biennial pattern has been described previously, with strong peaks of activity in late spring-summer months every second year (15).

Properties of seasonal coronaviruses

The duration of viral shedding was 5.6 days after experimental challenge with HCoV 229E in healthy volunteers (16).

Infection with HCoVs does not confer long-lasting sterile immunity. In a hospital- and community-based study of three coronaviruses (HCoV NL-63, OC-43, or 229E) among Kenyan children, 4-21% had documented re-infection within 47 and 98 days of the initial episode (17). In a serologic study from the Netherlands, ten healthy adults followed for 35 years experienced 101 infections (3 to 17 per individual) with HCoV NL-63, 229E, OC43, or

© 2021 Hawkes MT et al. *JAMA Network Open.*

HKU1, often repeatedly with the same strain (18). In an experimental challenge model with HCoV 229E in 15 healthy volunteers, 10 were infected, 8 were symptomatic, and 6 were infected a second time with repeat challenge 12 months later, although none of the second infections were symptomatic and the duration of viral shedding was reduced (16). Natural immunity to other severe and epidemic coronaviruses, SARS-CoV and MERS-CoV, has not been reported.

On the other hand, short-lived immunity and associated antibody responses are well documented. Volunteers resistant to initial HCoV 229E challenge had significantly higher levels of specific serum IgG and nasal IgA, suggesting that prior natural infection conferred temporary immunity (16). Although the precise duration of immunity is not known, these same volunteers were susceptible to re-infection after a period of 12 months (16). In another study, antibodies levels following natural infection decayed by 50% and 75% within 6 and 12 months of infection, respectively (18). In another modelling study, immunity to HCoV OC43 and HKU1 lasted approximately 45 weeks (19).

Properties of SARS-CoV-2

The basic reproduction number (R0) for SARS-CoV-2 (average number of new infections per infected at the beginning of the epidemic), has been estimated to be around R0 = 2 – 3 (2.2 with 90% high density interval 1.4–3.8 (20) or 2.7 with a 95% CrI of 2.47–2.86 (21).

Whether temporary or longer-term immunity to SARS-CoV-2 exists is unknown, but has important implications for natural and vaccine-induced immunity. Using a non-human primate model, resistance to re-infection with SARS-CoV-2 has been demonstrated one month after initial infection, with associated SARS-CoV-2-specific memory B-cells (22).

© 2021 Hawkes MT et al. *JAMA Network Open.*

PCR positivity in patients with COVID-19 can return following repeated negative results (23, 24); however, the relatively short period before repeat positivity suggests they may not have cleared the initial infection. Reinfection with SARS-CoV-2 several months after clearing an initial infection has been reported (25). It is thus too early to say whether natural immunity to SARS-CoV-2 will contribute to herd immunity.

© 2021 Hawkes MT et al. *JAMA Network Open.*

eAppendix 3. Cox Proportional Hazard Model for Time to First RSV Hospitalization

This analysis was based on data from the birth cohort of all infants born in the province of Alberta between 1 July 2005 and 30 June 2012. A time-to-event analysis (from birth to first hospitalization) was used to model RSV hospitalizations as a function of month and year of birth (Cox proportional hazard model).

We assumed that all hospitalizations in the birth cohort were included in the Alberta Health Services Discharge Abstract Database, which captures discharge diagnoses from all hospitals in the province. We included children born between 2005 and 2012, since 5 year follow-up data would be available for these children (data extracted for admissions from 2005 to 2017). The month and year of birth of all children in the province from 2005 to 2012 were available from publicly available vital statistics data (https://www150.statcan.gc.ca/t1/tbl1/en/cv.action?pid=1310041401).

The dependent (time-to-event) variable was the interval from birth to the age at first RSV admission. The independent variables were the month of the year (e.g., January, coded as a 12-level categorical variable) and the RSV season (even or odd, coded as a binary variable). Members of the birth cohort who were not hospitalized for RSV were censored at age 5 years.

© 2021 Hawkes MT et al. *JAMA Network Open.*

eAppendix 4. Qualitative Analysis of Model

To simplify the qualitative analysis, we assumed that the birth rate and death rate were equal, such that the population was of constant size. Without loss of generality, we assumed a population size N=1, such that S, I and R were interpreted as proportions of the total population. The *SIRS* model has two stationary solutions: a disease free equilibrium (*DFE*): (S, I, R) = (0, 0, 0) and an endemic equilibrium (*EE*): (S^*, I^*, R^*). The algebraic equations for the *EE* as a function of model parameters are:

$$S^* = \frac{\delta + \mu}{\beta}$$

$$I^* = \left(1 - \frac{\delta + \mu}{\beta}\right)\left(\frac{\gamma + \mu}{\delta + \gamma + \mu}\right)$$

$$R^* = \left(1 - \frac{\delta + \mu}{\beta}\right)\left(\frac{\delta}{\delta + \gamma + \mu}\right)$$

Seasonal forcing was introduced by letting the contact rate be a periodic function of the form (3, 27):

$$\beta(t) = b_0 \left[1 + b_1 \cos\left(\frac{2\pi t}{P} + \phi\right)\right]$$

When b_1 was small, the *SIRS* model had a periodic solution with period *T*. (eFigure 1). The basic reproductive number became a periodic function of time that oscillated between the values $\mathcal{R}_0^- = \frac{b_0(1-b_1)}{\delta+\mu}$ and $\mathcal{R}_0^+ = \frac{b_0(1+b_1)}{\delta+\mu}$. The endemic point also became a periodic function of time that moved back and forth between two extreme points, EE^- and EE^+. The limit cycle was asymptotically stable if $\mathcal{R}_0(t) > 1$ (27) (eFigure 1).

© 2021 Hawkes MT et al. *JAMA Network Open.*

When b_1 was larger $\left(b_1 > 1 - \frac{\delta+\mu}{b_0}\right)$, $\mathcal{R}_0$ could cross the boundary of 1 and the endemic point changed from stable to unstable during the period. This represents the scenario where seasonality was intense and a period-doubling bifurcation in the limit cycle occured (eFigure 1).

The following race analogy has been used to explain the model's solution (27). The trajectory can be thought of as a hopeless 'pursuer' in a race to catch the *EE*, which plays the role of the fast 'leader' that cannot be caught (27). While $\mathcal{R}_0(t) > 1$, the leader is deemed as stable *EE*. As soon as $\mathcal{R}_0(t) < 1$, the leader is deemed unstable and the solution's trajectory pursues the *DFE*. At that moment, the trajectory 'switches its objective,' pursues the *DFE* instead of the *EE*, and continues to do so while $\mathcal{R}_0(t) < 1$. The sudden change of objective gives rise to a period doubling bifurcation of the limit cycle (eFigure 1).

The stable limit cycle for the nonlinear *SIRS* model with seasonal forcing under realistic assumptions for model parameters showed a biennial pattern in the infectious and recovered fractions (eFigure 2).

Under what conditions will the stable limit cycle demonstrate a biennial pattern? We varied key model parameters b_1 and γ and determined the peak infected fraction (eFigure 3). As predicted by the bifurcation analysis, a biennial pattern emerged for $b_1 > 1 - \frac{\delta+\mu}{b_0} \approx 0.17$ (eFigure 3, Panel A). Furthermore, the biennial pattern was observed only over a range of values of duration of immunity: 160 days $< \frac{1}{\gamma} <$ 380 days (eFigure 3, Panel B). The following biological explanation may account for this observation. Short-lived immunity

© 2021 Hawkes MT et al. *JAMA Network Open.*

(<160 days) results in rapid decay of the recovered (immune) fraction before the onset of the next season. On the other hand, long-lasting immunity (>380 days) results in the accumulation of the recovered (immune) fraction over multiple years. When immunity wanes on the order of 1 year, a severe season results in a marked wave of infected individuals who enter the recovered (immune) fraction at a time coinciding with the following seasonal peak. The large fraction recovered (immune) individuals blunts the seasonal peak, generating the observed biennial pattern.

In summary, the following conditions were associated with a biennial seasonal pattern (numerical values were computed from plausible model parameters):

(1) The basic reproduction number of the model without seasonality must be greater than unity, in order for a non-zero stable endemic equilibrium to exist.

$$b_0 > \delta + \mu \approx 0.0986\ day^{-1}$$

(2) The seasonal variation must generate a period doubling bifurcation.

$$b_1 > 1 - \frac{\delta + \mu}{b_0} \approx 0.17$$

(3) The duration of immunity must be on the order of one year.

$$160\ days < \frac{1}{\gamma} < 380\ days$$

© 2021 Hawkes MT et al. *JAMA Network Open.*

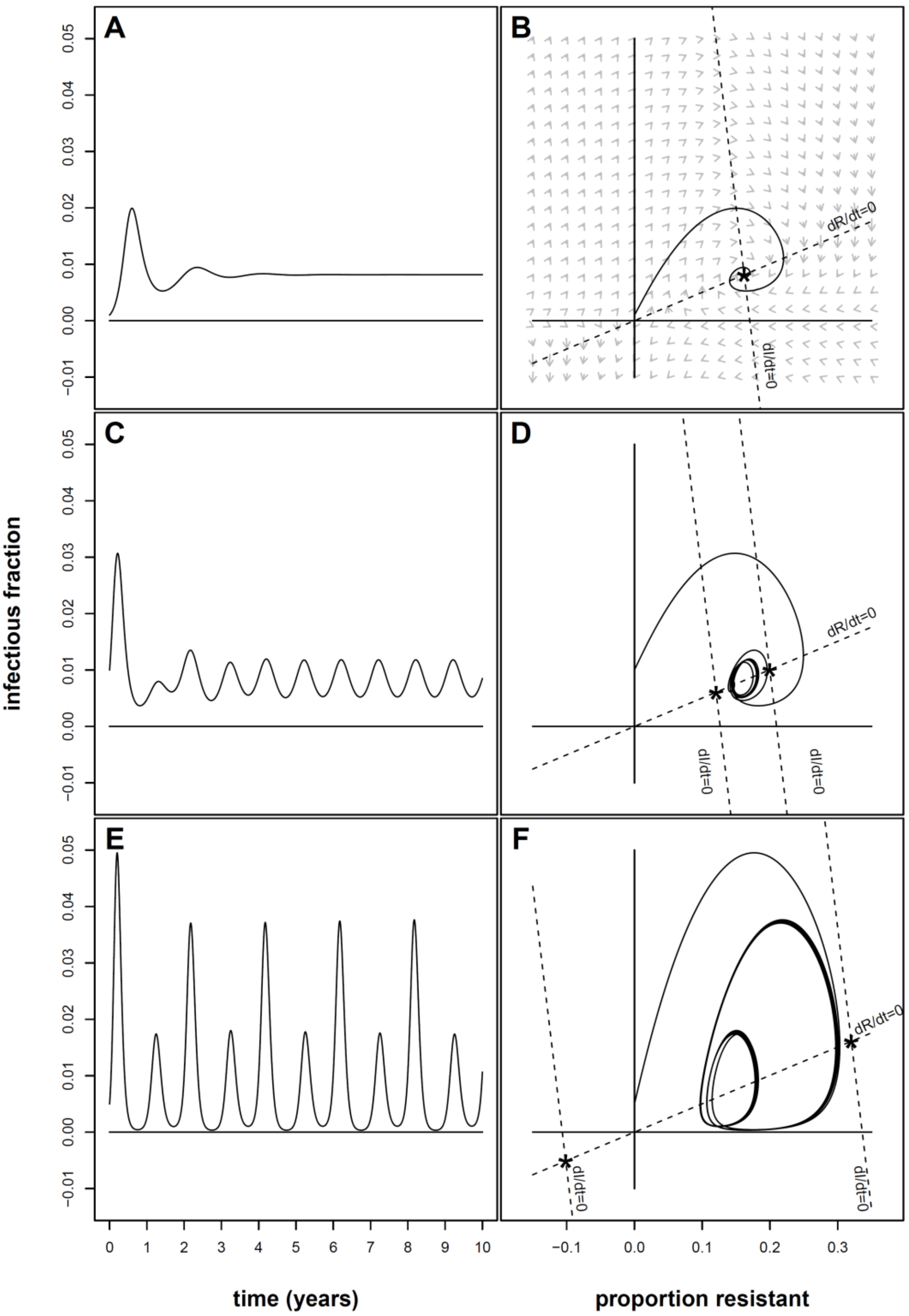

eFigure 1. *SIRS* Mathematical Model of Seasonal Respiratory Viruses.

Mathematical model predictions show the trajectory of a seasonal epidemic "seeded" at time t=0 with a small number of infected individuals. Over several years, a stable pattern emerged, depending on the seasonality. **A and B**. No seasonality. **A.** The disease trended toward an

© 2021 Hawkes MT et al. *JAMA Network Open.*

endemic equilibrium through several damped oscillations. **B.** In the I-R phase plane, the trajectory spiraled toward a stable steady state (star point *). Grey arrows in the phase portrait show the direction of the trajectory at any point (vector field). **C and D.** Mild seasonality (5%). **C.** Stable annual oscillations were eventually established. **D.** In the I-R phase plane, the trajectory trended toward a stable ovoid limit cycle. Of note, there was not a single point equilibrium, but a shifting attractor (line between star points *-*). **E and F**. High seasonality (25%), as in Edmonton, Canada. **E.** A more complex pattern emerged with biannual high and low peaks in the disease prevalence. **F.** The phase diagram showed large and small loops representing alternating years of high and low prevalence. Of note, the shifting equilibrium crossed the origin (I=0, R=0), at which point the equilibrium became unstable, and the disease temporarily trended toward extinction during this part of the year. This is the period doubling bifurcation.

© 2021 Hawkes MT et al. *JAMA Network Open.*

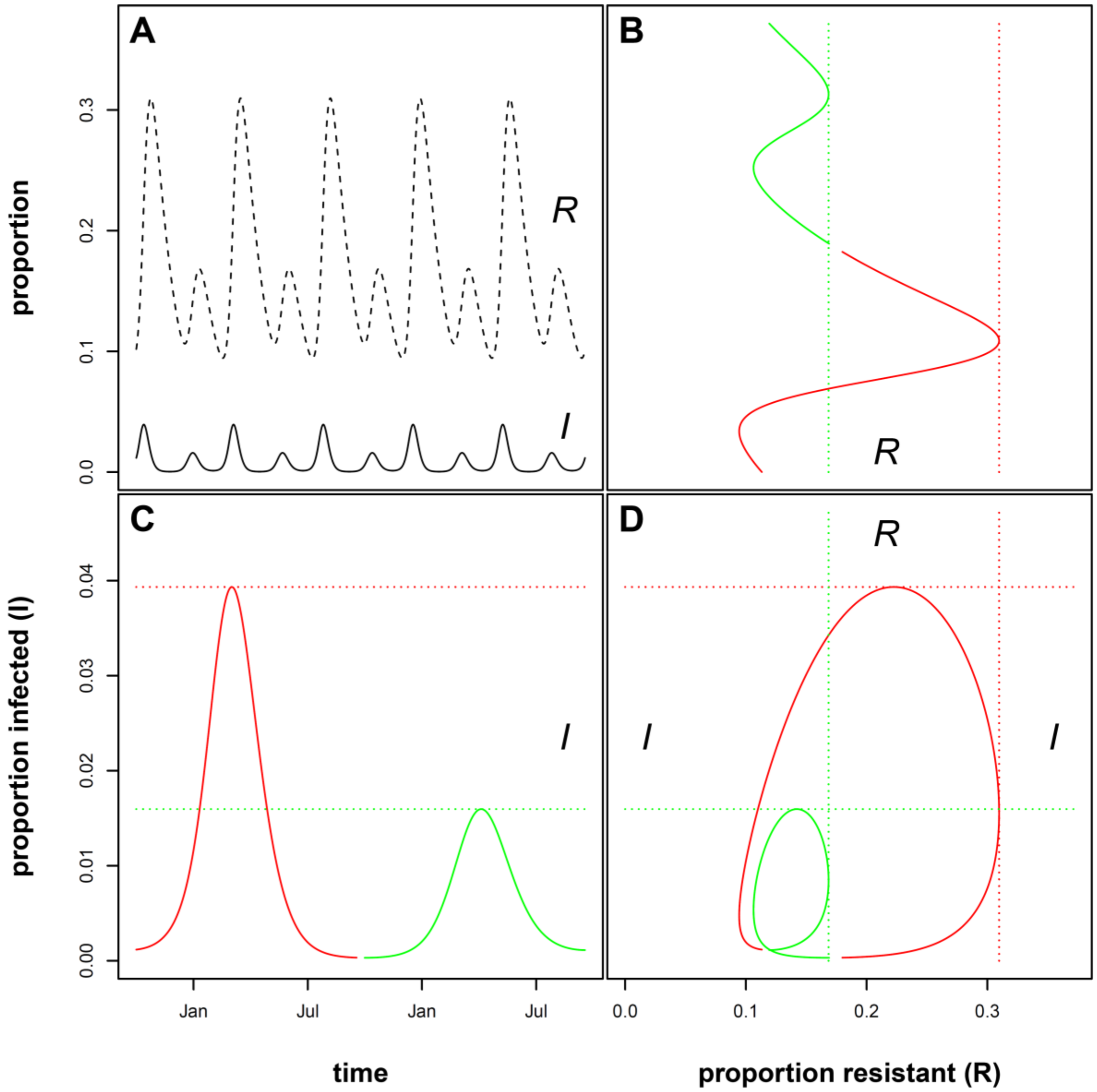

eFigure 2. Stable Biennial Limit Cycle.

A. Using fitted parameters from the data for Edmonton, Canada, and after allowing the model to stabilize (50 years run-in time), the proportion of infected (*I*) and resistant (*R*) individuals displayed a biennial pattern, shown over 10 years. **B**, **C, and D**. The R time series plotted vertically over two years **(B)** and the *I* time series plotted horizontally **(C)** with high (red) and low (green) prevalence years were mapped onto the *I*-*R* phase plane **(D)** to show the stable limit cycle with large (red) and small (green) annual loops.

© 2021 Hawkes MT et al. *JAMA Network Open.*

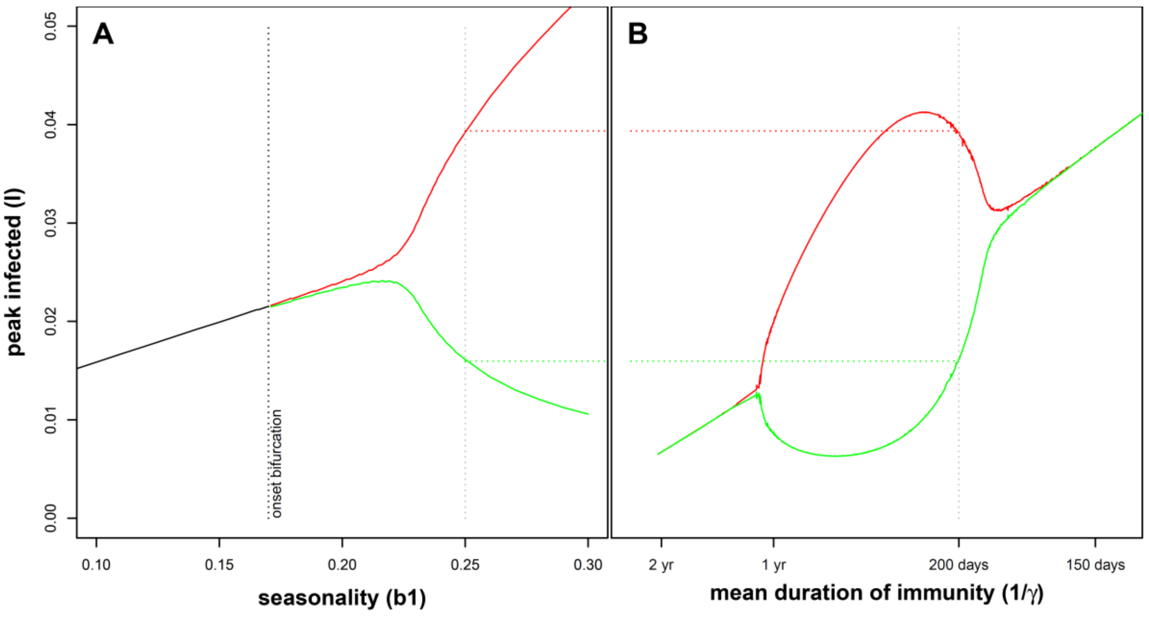

eFigure 3. Sensitivity Analysis and Bifurcation Diagrams, Showing Model Predictions With Different Seasonality (b_1) and Duration of Immunity (γ).

A. The parameter b_1 was varied from 0.1 (low seasonality) to 0.3 (high seasonality) and the stable limit cycles were analysed. With increasing b_1, the amplitude of the oscillations increases linearly up to $b_1 = 0.17$, where a period-doubling bifurcation occurs. Beyond this threshold, the local maxima in infected individuals split into high and low years. **B.** The parameter γ was varied from 0.001 days^{-1} (immunity lasts mean 1000 days) to 0.008 days^{-1} (immunity lasts mean 125 days). The greatest biennial difference occurs when the immunity wanes over a period of 6 to 12 months. For RSV, the mean duration of immunity was estimated to be 200 days. This may be understood as the effect of temporary population immunity spilling over into the next year, coinciding with the period of highest seasonal force of infection. A large "wave" of infection in year one generates immune individuals in year 2 which blunts the seasonal epidemic in year 2 but immunity does not extend into subsequent years.

© 2021 Hawkes MT et al. *JAMA Network Open.*

eTable. Model Parameters That Optimize the Fit Between SIRS Model and Observed Weekly Counts of Respiratory Viruses

	**RSV**	**hMPV**	**HCoV 229E**	**HCoV NL63**	**HCoV OC43**	**HCoV HKU1**
Scaling factor [$\times 10^{-5}$]	159	50.3	8.99	12.3	12.4	5.69
b_0 [day^{-1}]	0.121	0.121	0.121	0.120	0.121	0.125
b_1	0.246	0.249	0.237	0.245	0.222	0.373
ϕ [days]	-20	2	20	-18	-42	-18
δ [$\times 10^{-2}$ day^{-1}]	9.86	9.76	9.76	9.86	9.96	9.86
γ [$\times 10^{-3}$ day^{-1}]	4.88	4.69	4.93	4.93	3.95	4.55

© 2021 Hawkes MT et al. *JAMA Network Open.*

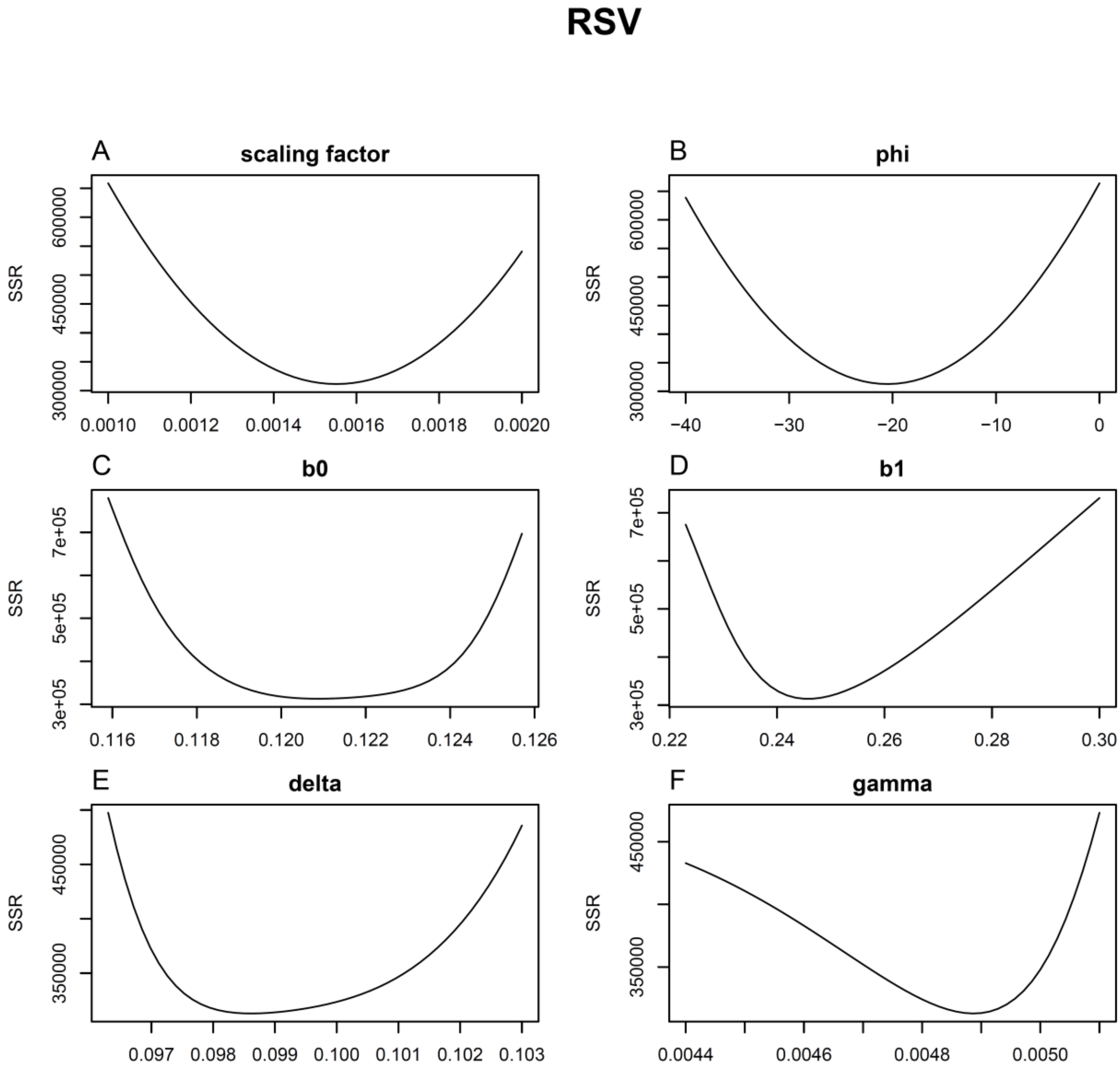

eFigure 4. Optimization of Model Parameters for RSV.

© 2021 Hawkes MT et al. *JAMA Network Open.*

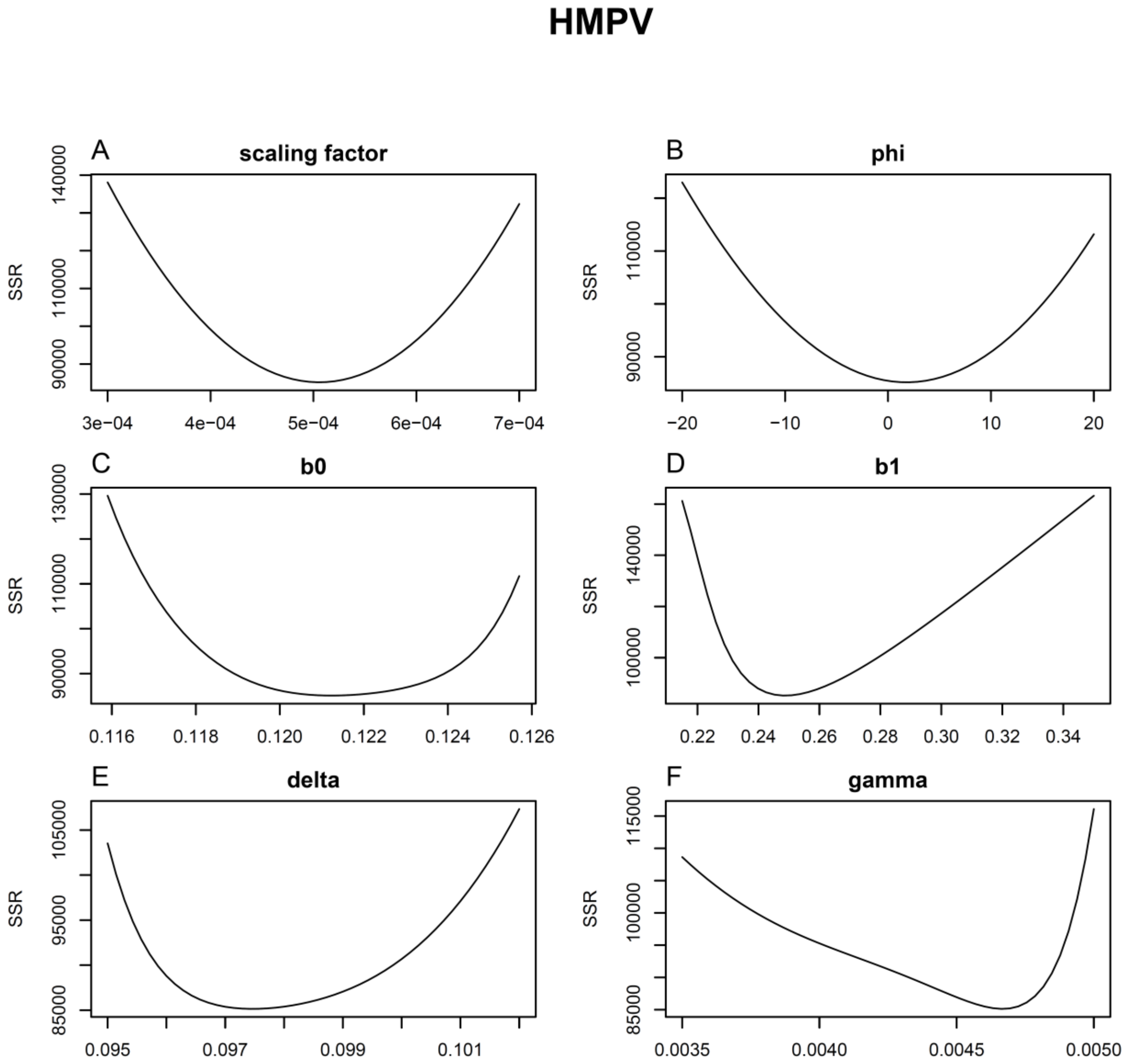

eFigure 5. Optimization of Model Parameters for hMPV.

© 2021 Hawkes MT et al. *JAMA Network Open.*

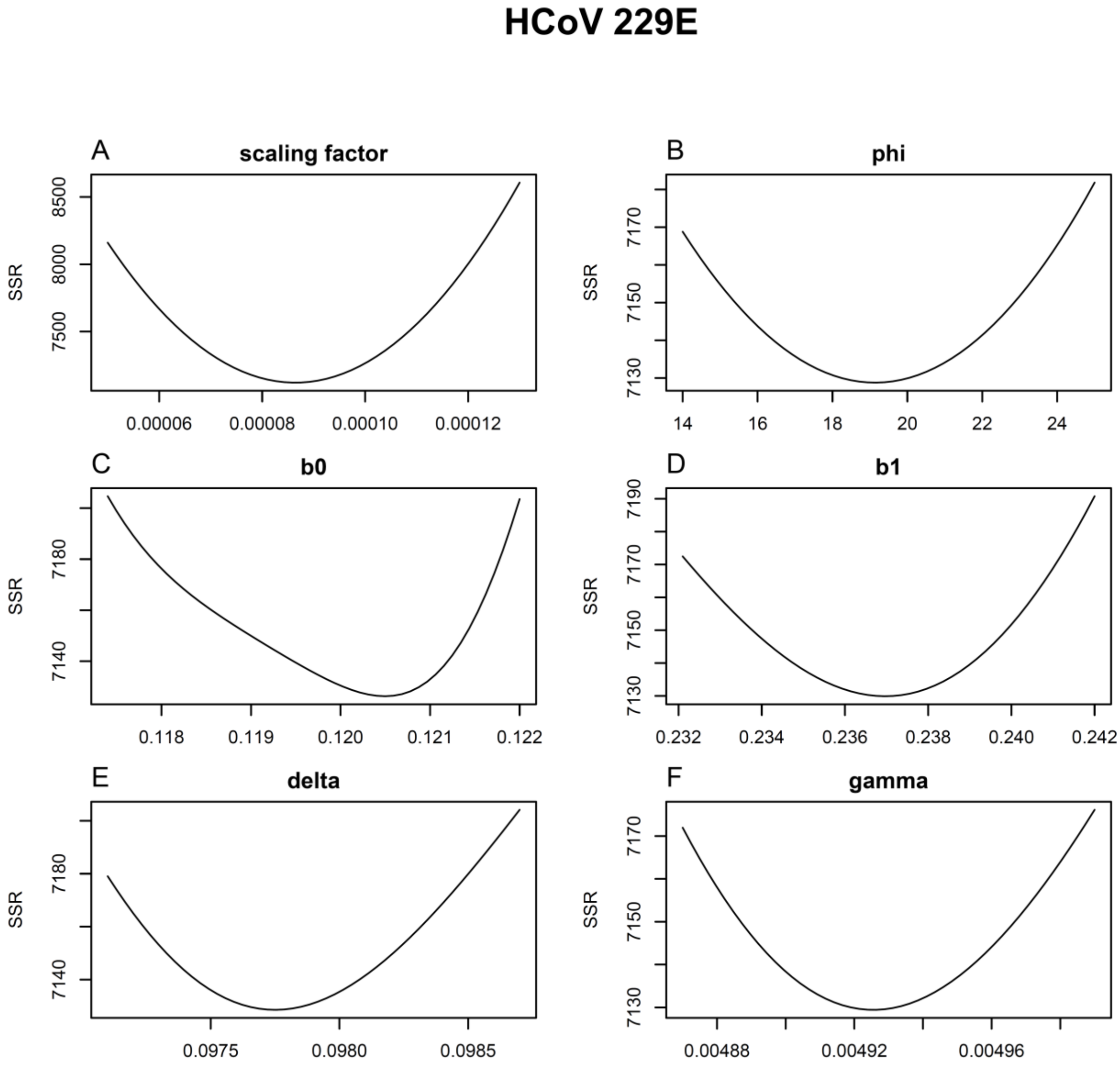

eFigure 6. Optimization of Model Parameters for HCoV 229E.

© 2021 Hawkes MT et al. *JAMA Network Open.*

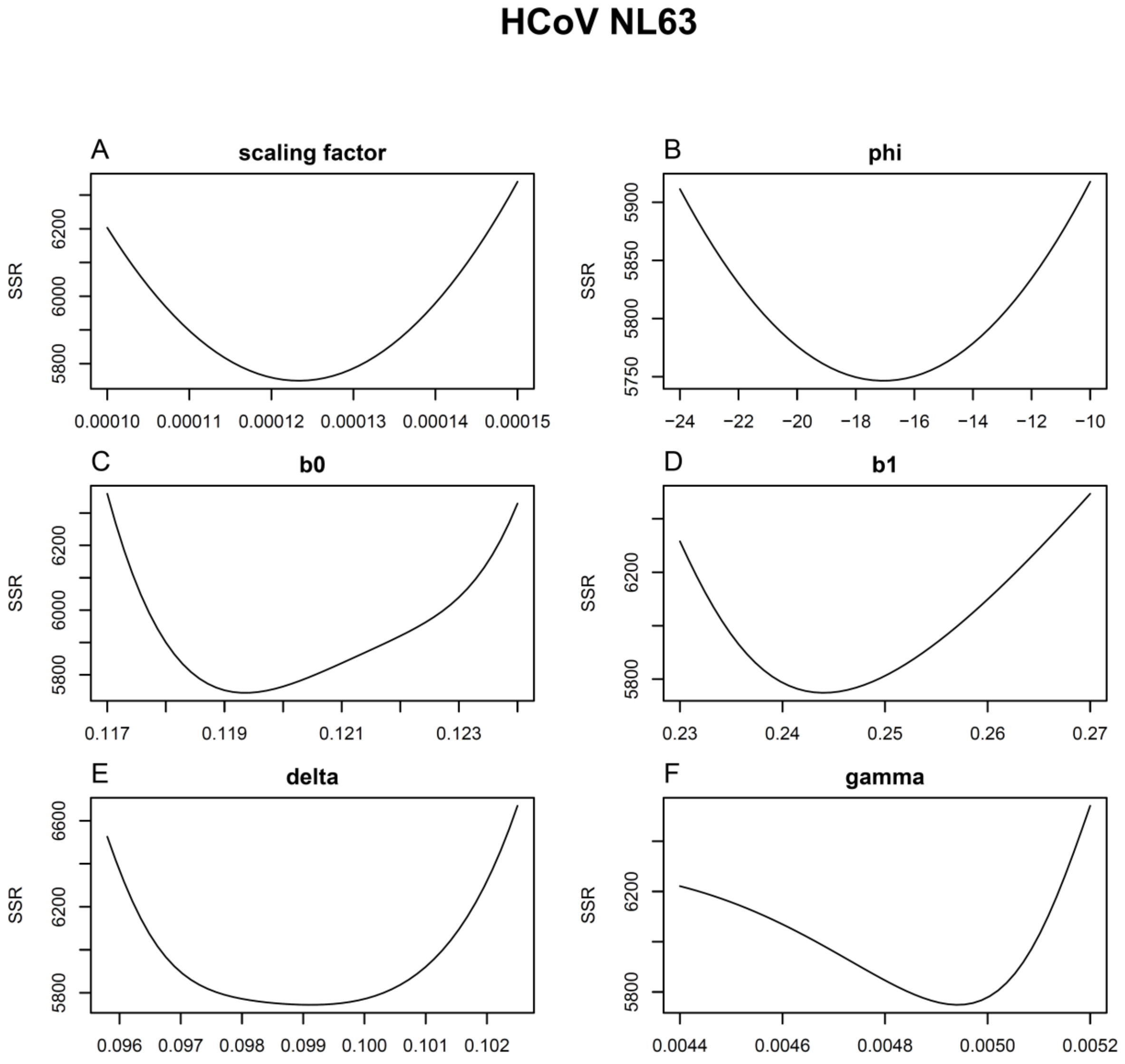

eFigure 7. Optimization of Model Parameters for HCoV NL63.

© 2021 Hawkes MT et al. *JAMA Network Open.*

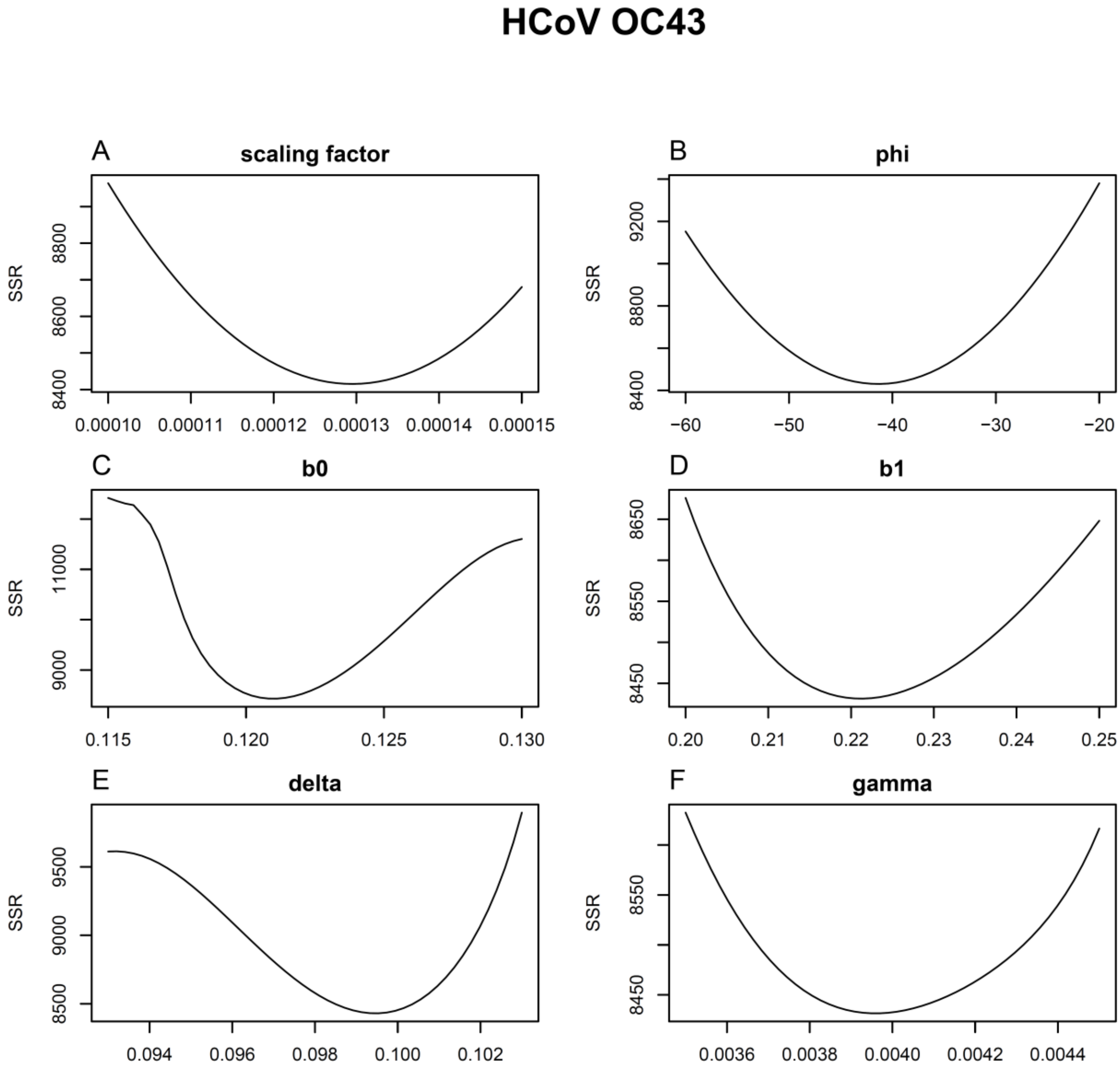

eFigure 8. Optimization of Model Parameters for HCoV OC43.

© 2021 Hawkes MT et al. *JAMA Network Open.*

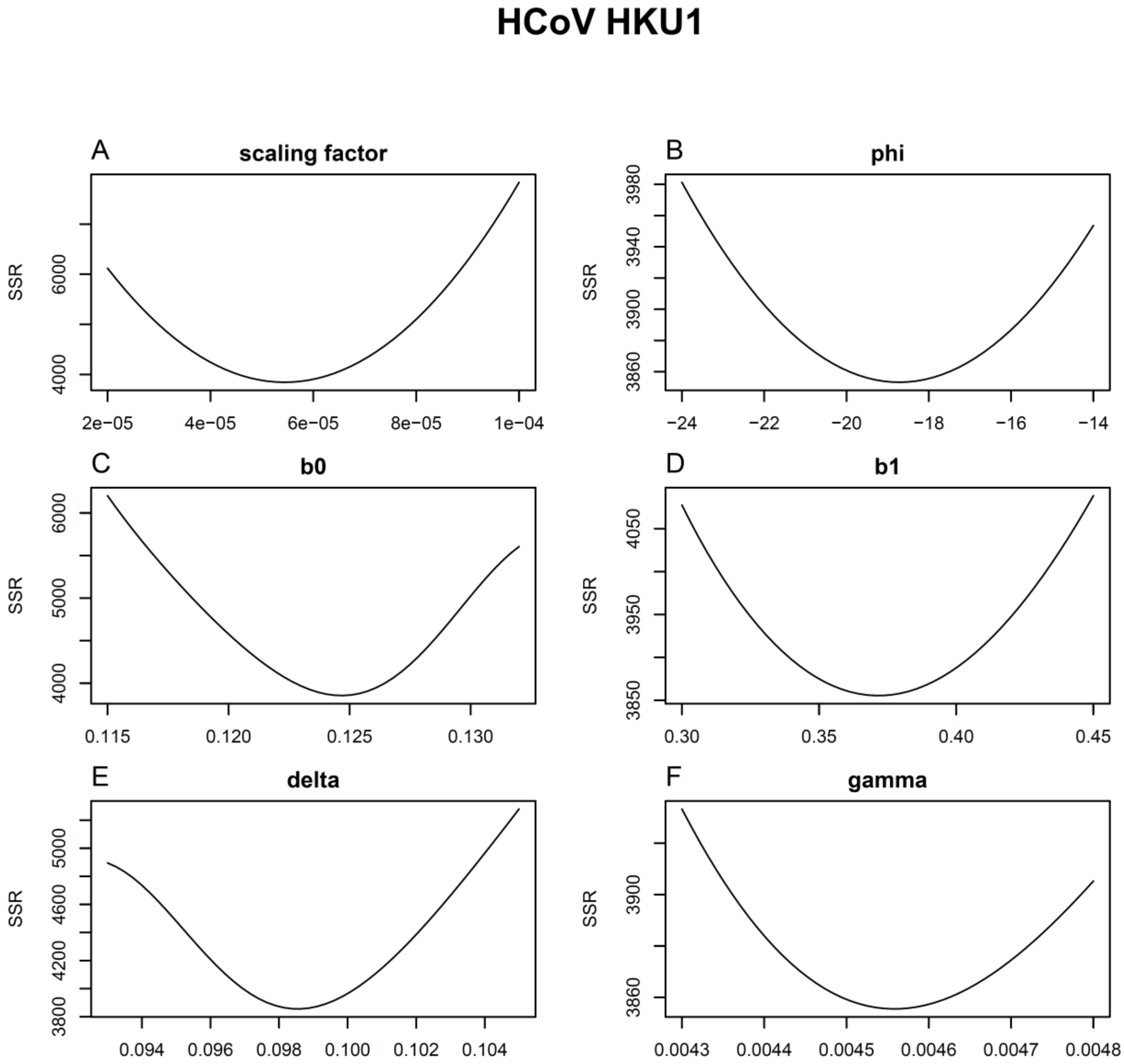

eFigure 9. Optimization of Model Parameters for HCoV HKU1.

© 2021 Hawkes MT et al. *JAMA Network Open.*

eReferences

1. Glezen WP, Taber LH, Frank AL, Kasel JA. Risk of primary infection and reinfection with respiratory syncytial virus. Am J Dis Child. 1986;140(6):543-6.
2. Wong K, Robinson JL, Hawkes MT. Risk of Repeated Admissions for Respiratory Syncytial Virus in a Cohort of >10 000 Hospitalized Children. J Pediatric Infect Dis Soc. 2020.
3. Weber A, Weber M, Milligan P. Modeling epidemics caused by respiratory syncytial virus (RSV). Math Biosci. 2001;172(2):95-113.
4. Weber MW, Mulholland EK, Greenwood BM. Respiratory syncytial virus infection in tropical and developing countries. Trop Med Int Health. 1998;3(4):268-80.
5. Hall CB, Douglas RG, Jr., Geiman JM. Respiratory syncytial virus infections in infants: quantitation and duration of shedding. J Pediatr. 1976;89(1):11-5.
6. Monto AS, Lim SK. The Tecumseh study of respiratory illness. 3. Incidence and periodicity of respiratory syncytial virus and Mycoplasma pneumoniae infections. Am J Epidemiol. 1971;94(3):290-301.
7. Hall CB, Walsh EE, Long CE, Schnabel KC. Immunity to and frequency of reinfection with respiratory syncytial virus. J Infect Dis. 1991;163(4):693-8.
8. Belshe RB, Van Voris LP, Mufson MA. Impact of viral respiratory diseases on infants and young children in a rural and urban area of southern West Virginia. Am J Epidemiol. 1983;117(4):467-74.
9. van den Hoogen BG, de Jong JC, Groen J, Kuiken T, de Groot R, Fouchier RA, et al. A newly discovered human pneumovirus isolated from young children with respiratory tract disease. Nat Med. 2001;7(6):719-24.
10. Ebihara T, Endo R, Kikuta H, Ishiguro N, Yoshioka M, Ma X, et al. Seroprevalence of human metapneumovirus in Japan. J Med Virol. 2003;70(2):281-3.
11. Pavlin JA, Hickey AC, Ulbrandt N, Chan YP, Endy TP, Boukhvalova MS, et al. Human metapneumovirus reinfection among children in Thailand determined by ELISA using purified soluble fusion protein. J Infect Dis. 2008;198(6):836-42.
12. Peret TC, Boivin G, Li Y, Couillard M, Humphrey C, Osterhaus AD, et al. Characterization of human metapneumoviruses isolated from patients in North America. J Infect Dis. 2002;185(11):1660-3.
13. Matsuzaki Y, Itagaki T, Abiko C, Aoki Y, Suto A, Mizuta K. Clinical impact of human metapneumovirus genotypes and genotype-specific seroprevalence in Yamagata, Japan. J Med Virol. 2008;80(6):1084-9.
14. van den Hoogen BG, Herfst S, de Graaf M, Sprong L, van Lavieren R, van Amerongen G, et al. Experimental infection of macaques with human metapneumovirus induces transient protective immunity. J Gen Virol. 2007;88(Pt 4):1251-9.
15. Aberle JH, Aberle SW, Redlberger-Fritz M, Sandhofer MJ, Popow-Kraupp T. Human metapneumovirus subgroup changes and seasonality during epidemics. Pediatr Infect Dis J. 2010;29(11):1016-8.
16. Callow KA, Parry HF, Sergeant M, Tyrrell DA. The time course of the immune response to experimental coronavirus infection of man. Epidemiol Infect. 1990;105(2):435-46.
17. Kiyuka PK, Agoti CN, Munywoki PK, Njeru R, Bett A, Otieno JR, et al. Human Coronavirus NL63 Molecular Epidemiology and Evolutionary Patterns in Rural Coastal Kenya. J Infect Dis. 2018;217(11):1728-39.
18. Edridge AWD, Kaczorowska, J.M., Hoste, A.C.R., Bakker, M., Klein, M., Jebbink, M.F. et al. Coronavirus protective immunity is short-lasting. medRxiv. 2020.

© 2021 Hawkes MT et al. *JAMA Network Open.*

19. Kissler SM, Tedijanto C, Goldstein E, Grad YH, Lipsitch M. Projecting the transmission dynamics of SARS-CoV-2 through the postpandemic period. Science. 2020;368(6493):860-8.
20. Riou J, Althaus CL. Pattern of early human-to-human transmission of Wuhan 2019 novel coronavirus (2019-nCoV), December 2019 to January 2020. Euro Surveill. 2020;25(4).
21. Wu JT, Leung K, Leung GM. Nowcasting and forecasting the potential domestic and international spread of the 2019-nCoV outbreak originating in Wuhan, China: a modelling study. Lancet. 2020;395(10225):689-97.
22. Chandrashekar A, Liu J, Martinot AJ, McMahan K, Mercado NB, Peter L, et al. SARS-CoV-2 infection protects against rechallenge in rhesus macaques. Science. 2020.
23. Zheng KI, Wang XB, Jin XH, Liu WY, Gao F, Chen YP, et al. A Case Series of Recurrent Viral RNA Positivity in Recovered COVID-19 Chinese Patients. J Gen Intern Med. 2020.
24. Ye G, Pan Z, Pan Y, Deng Q, Chen L, Li J, et al. Clinical characteristics of severe acute respiratory syndrome coronavirus 2 reactivation. J Infect. 2020;80(5):e14-e7.
25. To KK, Hung IF, Ip JD, Chu AW, Chan WM, Tam AR, et al. COVID-19 re-infection by a phylogenetically distinct SARS-coronavirus-2 strain confirmed by whole genome sequencing. Clin Infect Dis. 2020.
26. Lee BE, Mukhi SN, May-Hadford J, Plitt S, Louie M, Drews SJ. Determination of the relative economic impact of different molecular-based laboratory algorithms for respiratory viral pathogen detection, including Pandemic (H1N1), using a secure web based platform. Virol J. 2011;8:277.
27. Ponciano JM, Capistran MA. First principles modeling of nonlinear incidence rates in seasonal epidemics. PLoS Comput Biol. 2011;7(2):e1001079.

© 2021 Hawkes MT et al. *JAMA Network Open.*
